# A preliminary study of schema therapy for young adults with high-functioning autism spectrum disorder: a single-arm, uncontrolled trial

**DOI:** 10.1186/s13104-021-05556-1

**Published:** 2021-04-29

**Authors:** Fumiyo Oshima, Tomokazu Murata, Toshiyuki Ohtani, Mikuko Seto, Eiji Shimizu

**Affiliations:** 1 Research Center for Child Mental Development, Chiba University, 1-8-1 Inohana, Chuouku, Chiba 260-8670 Japan; 2 Safety and Health Organization, Chiba University, Chiba, Japan

**Keywords:** Autism spectrum disorder, Schema therapy, Social adjustment, Quality of life

## Abstract

**Objective:**

Psychological problems associated with isolation and mistrust are common among young adults with autism spectrum disorder (ASD). Schema therapy (ST) has recently been shown to be effective against chronic personality problems of various mental disorders, including personality disorders. This pilot clinical trial aimed to explore the feasibility and acceptability of ST in young adults with high-functioning ASD.

**Results:**

Following the intervention, a significant reduction in early maladaptive schemas and improvements in quality of life and social adjustment were observed. ST may be feasible and is applicable to young adults with HF-ASD.

*Trial registration* UMIN000014535; registered on July 11, 2014

**Supplementary Information:**

The online version contains supplementary material available at 10.1186/s13104-021-05556-1.

## Introduction

Autism spectrum disorder (ASD) is a neurodevelopmental disorder with persistent deficits in social communication and social interaction based on the *Diagnostic and Statistical Manual of Mental Disorders, Fifth Edition* (DSM-5) [1]. ASD adults tend to have difficulties with social functioning, contributing to a poor quality of life (QOL) [2]. ASD children diagnosed before the age of 10 years tend to have a higher psychosocial QOL than those who are not [3].

Unfortunately, most high-functioning ASD (HF-ASD) individuals remain undiagnosed at early ages because of high functioning [4], and they do not receive appropriate support, as symptoms remain unnoticed for the same reason [5, 6]. Unawareness of their ASD traits could result in an inferiority complex and self-stigmatization, which may increase depression and anxiety [7].

Cognitive behavioral therapy (CBT) is frequently used to treat ASD related symptoms. A meta-analytic review revealed that cognitive behavioral therapy effectively treats anxiety in children with ASD [8]. However, the effects of cognitive-behavioral therapy on depression in children and adults [9] and anxiety [10, 11] in adults with ASD are inconsistent. Several studies have shown that ASD after puberty is characterized by strong self-stigmatization due to chronic social isolation [12]. Such a self-stigma corresponds to an early maladaptive schema (EMS) in terms of schema therapy. It is known that patients with ASD have significantly higher EMS than healthy people, resulting in a negative impact on mental health [13]. Since CBT is a symptom-specific treatment, it does not focus on EMS. Therefore, improving CBT for ASD individuals is clinically important.

Schema therapy (ST) [14], an innovative and integrative psychotherapy model, is used to treat those who face difficulties with personality disorder diagnoses [15–20]. ST is composed of an EMS, schema mode (SM), limited reparenting, and core emotional needs [21, 22]. EMS is an excessively generalized cognitive and emotional system composed of innate features and early childhood experiences. When an individuals’ core needs are unfulfilled in early childhood, they can form multiple EMS, which develop a SM that temporarily comes to the fore and dominates their presentation, resulting in difficulties in interpersonal relationships [23]. We previously confirmed that HF-ASD individuals have more EMS than general controls [24]. As EMS are assumed to be the core of the psychopathology of HF-ASD and personality disorders, learning how to change EMS into more adaptive ones, and utilizing their adaptive coping responses, is the ultimate goal of ST [25]. The purpose of ST is to cultivate a more constructive “healthy adult mode” (instead of schema mode) toward environmental stimuli by gratifying patients’ core needs using “limited reparenting.” ST, a structured type of psychotherapy, is effective [15, 26, 27] and suitable for many psychiatric disorders; HF-ASD adults are considered suitable for ST, as they generally benefit from structured settings [28]. In this study, we identified EMSs specific to HF-ASD individuals [24]. However, studies on the clinical feasibility and acceptability of ST for HF-ASD are limited. Thus, this pilot study used ST for adults with HF-ASD and examined its feasibility and acceptability in improving patients’ EMS and SM and the consequent improvements in QOL and social adjustment.

## Main text

### Materials and methods

This was a single-arm preliminary study with an open trial design in a Japanese clinical setting.

#### Participants

Participants (n = 13) were recruited through psychiatrist referral at the Safety and Health Organization, Chiba University, and Chiba University Hospital, Chiba, Japan. The inclusion criteria were as follows: age between 18 and 40 years, intelligence quotient (IQ) ≥ 80 (Wechsler Adult Intelligence Scale III [29]), and an ASD diagnosis based on the Autism Diagnostic Interview-Revised [30] and/or the Autism Diagnostic Observation Schedule—Second Edition [31]. The Mini-International Neuropsychiatric Interview [32] was used to evaluate comorbid psychiatric conditions. The exclusion criteria were: a history of substance abuse, active suicidality, and severe mental and physical conditions. Participants were adults of average intelligence with ASD who could respond on their own initiative after understanding the reason for obtaining research consent.

#### Procedures

**Fig. 1:**
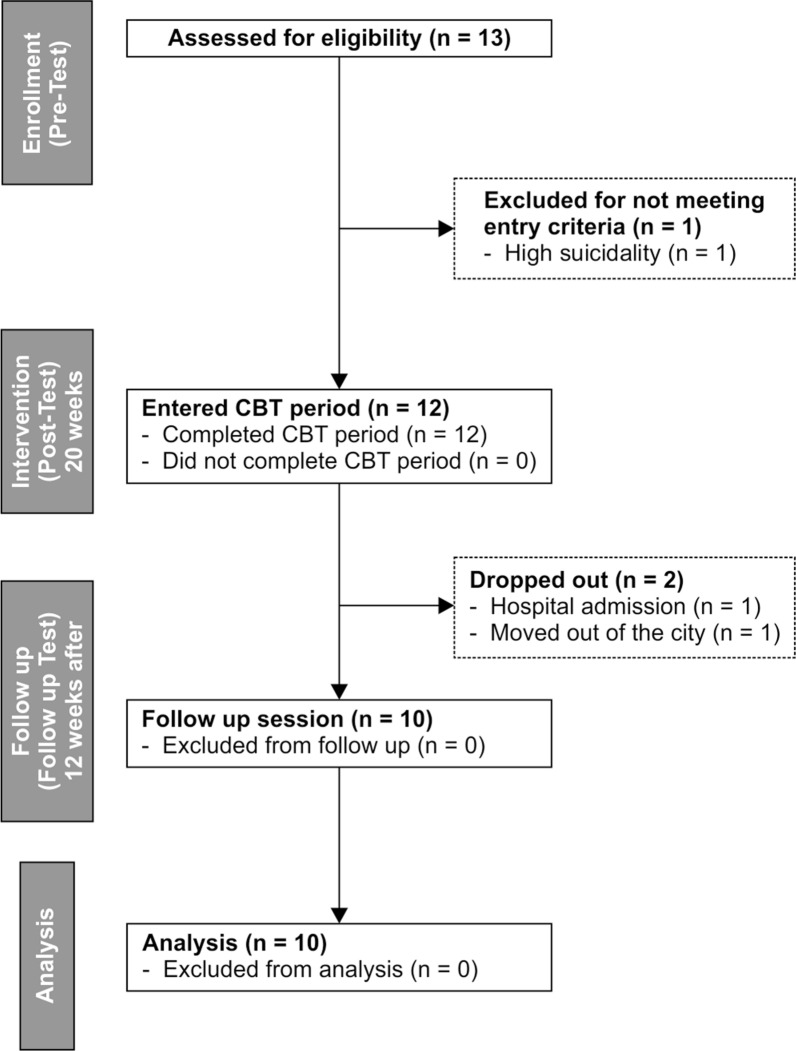
CONSORT flow chart of the clinical trial

Recruitment, treatment, and data collection were conducted between September 2014 and March 2018; the trial flow chart is shown in Fig. 1. Each weekly session lasted 50 min (Additional file 1: Table S1). A follow-up interview was conducted 12 weeks after the intervention completion (37th week).

#### Treatment outcome

The primary outcome measures were the Global Assessment Functioning (GAF) Scale [33], an interview rating of social functioning and the World Health Organization quality of life assessment brief (WHO QOL-BREF) [34], which was a self-rating scale measuring subjective feeling of social adaptiveness and QOL. The secondary outcomes were the scores of the Young Schema Questionnaire-Short Form 3 (YSQ-SF) [35] and the Schema Mode Inventory (SMI) [36], which measure EMS and the schema modes of patients, respectively. Other outcome variables of comorbid symptoms, such as depression, anxiety, and obsessive–compulsive symptoms, were assessed by the Beck Depression Inventory II (BDI-II) [37], the State-Trait Anxiety Inventory (STAI) [38], Liebowitz Social Anxiety Scale (LSAS) [39], and Obsessive–Compulsive Inventory [40], respectively. Permission was not needed to use these questionnaires.

#### Analysis

Data were analyzed using repeated-measures analysis of variance (rANOVA) with time as the independent variable. In addition, a paired t-test adjusted by a Bonferroni correction was used for pairwise comparisons. For the primary outcomes and other comorbid symptom measurements, the adjusted α value was α = 0.05/3/9 = 0.002. For the secondary outcomes, the adjusted α value was 0.05/3/23 = 0.0007 for YSQ-SF and 0.05/3/16 = 0.001 for SMI. Effect sizes were calculated for changes in scale scores between time points (Cohen’s *d*). All analyses were conducted using SPSS for Windows version 23 (IBM, Armonk, NY, USA).

### Results

#### Patients’ demographics and baseline data

Of the 13 patients, one was excluded, and two dropped out of the study, leaving 10 patients (Fig. 1). The participants were aged between 20 and 39 years and met the DSM-5 criteria for ASD (Table 1 and Additional file 2: Table S2).

**Table 1:** Baseline demographic and clinical characteristics

Variable	Mean (SD)/frequency (%)
Age	26.8 (6.39)
Sex	
Female	5 (50%)
Male	5 (50%)
Education	
Master	2 (20%)
Bachelor	7 (70%)
High school	1 (10%)
Employment	
Student	7 (70%)
Part timer	1 (10%)
Unemployed	2 (20%)
Diagnosis	
ASD + OCD	4 (40%)
ASD + ADHD	1 (10%)
ASD + DEP	5 (50%)
ADI-R	
Quality of reciprocal social interaction	16 (4.22)
Communication	13.3 (4.37)
Repetitive, restricted, and stereotyped patterns of behavior	3.2 (1.75)
Abnormalities of behavior evident at or before 36 months	1.8 (1.14)
ADOS-2	
Communication	3.8 (1.48)
Reciprocal social interaction	7 (2.54)
Imagination/creativity	1.4 (0.52)
Restricted and repetitive behaviors	1.2 (0.63)
WAIS-3	
VIQ	120.8 (10.6)
PIQ	117.7 (11.04)
FIQ	120 (9.51)
VC	119.7 (12.31)
PO	120 (12.75)
WM	112.6 (11.16)
PS	109.7 (19.29)

*ADHD* attention deficit hyperactivity disorder, *ADI-R* Autism Diagnostic Interview-Revised, *ADOS-2* Autism Diagnostic Observation Schedule-2, *DEP* depression, *FIQ* full scale IQ, *OCD* obsessive–compulsive disorder, *PIQ* performance IQ, *PO* perceptual organization, *PS* perceptual organization, *VC* verbal comprehension, *VIQ* verbal IQ, *WAIS-3* The Wechsler Adult Intelligence Scale-3, *WM* working memory

#### Treatment outcomes

The rANOVA revealed a significant main effect of time on the primary outcome, GAF. A post hoc analysis revealed that there were significant differences between pre-and post-treatment (*p* < 0.001, *d* = 3.30) and between pre-treatment and follow-up (*p* < 0.001, *d* = 3.35) (Table 2). Additionally, the main effect of time for WHO QOL-BREF was significant; however, the post hoc analyses results did not remain significant between each time point after the correction.

**Table 2:** Changes in each evaluated item before ST, after ST, and at follow-up

Variable	Pre-ST	Post-ST	Follow-up	*F*	Cohen's *d*		
	Mean (SD)	Mean (SD)	Mean (SD)		Pre-ST vs. post-ST	Post-ST vs. follow-up	Pre-ST vs. follow-up
GAF	45.90 (10.82)	76.00 (7.06)^a^	76.20 (6.83)^c^	*F* (1.3, 11.67) = 151.65**	3.30	0.03	3.35
QOL	65.20 (16.03)	75.70 (14.28) ^a^	78.50 (12.77)	*F* (1.21, 10.87) = 9.93**	0.69	0.21	0.92
BDI-II	27.70 (17.54)	16.70 (13.94)	13.00 (11.76)	*F* (1.16, 10.4) = 9.64**	0.70	0.29	0.99
STAI (state)	45.20 (9.47)	49.70 (8.21)	41.50 (6.92)^b^	*F* (2, 18) = 3.93*	0.51	1.08	0.45
STAI (trait)	55.80 (14.16)	53.00 (10.53)	47.50 (8.82)	*F* (2, 18) = 3.56*	0.23	0.57	0.70
OCI	67.70 (30.94)	59.40 (27.39)	49.70 (23.15)	*F* (1.17, 10.49) = 3.98	0.29	0.38	0.66
LSAS_Total	62.70 (22.60)	62.80 (27.53)	50.70 (18.64)	*F* (1.2, 10.81) = 2.71	0.00	0.52	0.58
LSAS_fear/anxiety	35.90 (13.54)	35.40 (15.18)	27.20 (9.32)	*F* (2, 18) = 5.22*	0.04	0.65	0.75
LSAS_escape	26.80 (13.23)	27.40 (13.70)	23.50 (11.21)	*F* (1.27, 11.46) = 0.59	0.05	0.31	0.27

BDI-II, Beck Depression Inventory-II; LSAS (Total score), Total items from the Liebowitz Social Anxiety Scale; LSAS (Fear/Anxiety), Fear and anxiety items from the Liebowitz Social Anxiety Scale; LSAS (Avoidance), Avoidance items from the Liebowitz Social Anxiety Scale; STAI (State), State items from the State-Trait Anxiety Inventory; STAI (Trait), Trait items from the State-Trait Anxiety Inventory; OCI, Obsessive Compulsive Inventory

**p < 0.01, *p < 0.05 calculated using analysis of variance

^a^Significant difference between pre- and post-intervention values (p < 0.05, Bonferroni corrected)

^b^Significant difference between post-intervention and follow-up values

^c^Significant difference between pre-intervention and follow-up values (p < 0.05, Bonferroni corrected)

Regarding the secondary outcomes, the rANOVA revealed a significant effect of time on the YSQ-SF (total score), disconnection and rejection, impaired autonomy and performance, impaired limits (over-vigilance and inhibition), social isolation/alienation schema, failure schema, dependence/incompetence schema, emotional inhibition schema, approval-seeking/recognition-seeking schema, negativity/pessimism schema, and punitiveness schema scores, but not on other subscales (Additional file 3: Table S3). The Bonferroni post hoc test showed a significant difference between pre-treatment and the follow-up (*p* = 0.00048, *d* = 1.01) for the YSQ-SF (total score). Similarly, for disconnection and rejection, a significant difference was found between post-treatment and follow-up (*p* = 0.00026, *d* = 0.34). There was also a significant difference for impaired limits (over-vigilance and inhibition) between pre-treatment and follow-up (*p* = 0.00002, *d* = 0.63). In addition, for the emotional inhibition schema and the negativity/pessimism schema, although the ANOVA showed a significant main effect of time, the Bonferroni post hoc test did not show any significance.

Regarding the SMI, the repeated-measures ANOVA revealed a significant main effect of time for the scores of the adaptive, maladaptive, vulnerable child, angry child, enraged child, impulsive child, undisciplined child, happy child, compliant surrender, detached protector, self-aggrandizer, punitive parent, and demanding parent, and healthy adult modes (Additional file 4: Table S4).

Conversely, there was no significant difference in the detached self-soothing or bullying and attack modes. Bonferroni post hoc tests showed a significant difference between pre-treatment and follow-up for the maladaptive (*p* = 0.00051, *d* = 1.57), enraged child, (*p* = 0.00012, *d* = 1.86), undisciplined child (*p* = 0.00066, *d* = 0.97), and demanding parent modes (*p* = 0.00010, *d* = 1.10).

Regarding other outcomes, significant effects of time were observed for BDI-II, STAI-state and -trait, and LSAS fear/anxiety; however, post hoc comparison with Bonferroni correction did not remain significant between each time point.

### Discussion

This study examined the feasibility and acceptability of ST for HF-ASD adults. Furthermore, this study provided a proof-of-concept where adults with high-functioning ASD had improved QOL and social functioning after receiving ST. ST aims to improve chronic psychological maladjustment rather than specific symptoms of individuals. The psychological maladjustment of adults with ASD includes isolation, which is anxiety regarding exclusion, and at a behavioral level, withdrawal and avoidance are common [5]. ST does not encompass the element of improving communication skills, but it may restore a sense of trust in others, which is the foundation of communication. For example, at the post-intervention, seven patients indicated social participation, such as returning to school or removing themselves from self-imposed isolation and receiving employment support.

This study showed a reduction in the YSQ total score, disconnection and rejection subscale, and impaired limits subscale. Moreover, ST for HF-ASD may have improved an individuals’ disconnected feelings towards others or the social environment and enhanced their self-control abilities. Regarding the subscales of EMS, individuals with ASD scored higher than individuals without social isolation, failure, and dependence-incompetence [29]. The results of this study showed that these three EMS (social isolation, failure, and dependence-incompetence) were significantly reduced after the intervention. Moreover, they are considered to influence communication style and social relationships [29], and improvement in EMS and social functioning may be important ST outcomes for HF-ASD. Additionally, we found that ST for HF-ASD showed a significant improvement in several subscales of SMI. Interestingly, these changes were statistically significant only from pre-intervention to follow-up, not from pre- to post-intervention. Since the schema mode is more stable than EMS, changes in the former may have delayed the effects. When considering the underlying mechanisms of delayed improvement in SMI, decreased scores in the enraged child, undisciplined child, and demanding parent mode may have reduced individual irritability and aggressive behavior, leading to less social interaction-related problems. Moreover, the maladaptive coping mode can enhance avoidance behaviors and/or over-adaptations, and the reduced maladaptive coping mode via ST may enable individuals to participate socially.

ST targets EMS at the deepest cognition level and is considered better equipped to enable dysfunctional schematic processing changes. For example, ST showed clinical efficacy in treatment-resistant patients, such as those with personality disorders [41]. It has been reported that some adult patients with ASD have a self-stigma against ASD and are highly resistant to treatment [5]. ST may enhance ASD adults’ awareness and acceptance of their ASD, which may also influence their social adaptiveness. Finally, we did not observe any significant improvement in comorbid symptoms after ST. Since baseline scores for those measurements were not high compared with patients with major depressive, anxiety, and obsessive–compulsive disorders, those with comorbid symptoms may not have improved.

## Conclusion

This study highlights that ST may be feasible for ASD participants and is applicable to individuals with HF-ASD. Additional data is required to ensure the clinical benefit of ST for individuals with HF-ASD. Future clinical trials should incorporate the following: (a) RCTs should be conducted to provide evidence of the effects of ST on adults with ASD, (b) homogeneity of the target population should be ensured by reducing the number of juxtaposed psychiatric disorders to one, and (c) not only self-rating scales but also other-rating indicators should be incorporated into the evaluation.

## Limitations

There are several limitations to this study. First, this study’s sample size was small and had some bias in the demographics of the subjects (mental disorders other than ASD, IQ, and age). Therefore, the results might have been biased and should be considered carefully.

Second, the study design should be improved from a single-arm to a randomized control design. This single-arm study did not incorporate controls during the secondary diagnoses, thereby restricting the comparability of this study with other psychological interventions in terms of the clinical efficacy of ST on social adaptations. Finally, it may be difficult to exclusively determine the efficacy of ST on social adaptiveness from this study. Since ST involves psychoeducation regarding ASD traits, the component of psychoeducation may influence social adaptiveness along with ST.

## Supplementary Information

**Additional file 1: Table S1**. Contents of schema therapy (ST) for high-functioning autism spectrum disorder (ASD).

**Additional file 2: Table S2**. Sociodemographic data.

**Additional file 3: Table S3**. Changes in Scores of Young Schema Questionnaire (YSQ) before and after ST at follow-up.

**Additional file 4: Table S4**. Changes in Schema Mode Inventory (SMI) before and after ST at follow-up.

## Data Availability

The datasets used and/or analyzed during the current study are available from the corresponding author on reasonable request.

## Abbreviations

ASD: Autism spectrum disorder
BDI-II: Beck Depression Inventory II
CBT: Cognitive-behavioral therapy
DSM-5: Diagnostic and statistical manual of mental disorders
EMS: Early maladaptive schema
GAF: Global assessment functioning
HAM: Healthy adult mode
HF-ASD: High-functioning autism spectrum disorder
IQ: Intelligence quotient
RCTs: Randomized control trial
QOL: Quality of life
SMI: Schema Mode Inventory
ST: Schema therapy
WHO QOL-BREF: World Health Organization Quality Of Life Assessment Brief
YSQ-SF: Young Schema Questionnaire Short Form 3
