# Supplementary material for: A preliminary study of schema therapy for young adults with high-functioning autism spectrum disorder: a single-arm, uncontrolled trial
Source: BMC Res Notes. 2021 Apr 29;14:158. doi: 10.1186/s13104-021-05556-1 (PMC8082897; doi:10.1186/s13104-021-05556-1)
Supplement: Supplementary file 1 — Additional file 1: Table S1. Contents of schema therapy (ST) for high-functioning autism spectrum disorder (ASD). [file 13104_2021_5556_MOESM1_ESM.docx]

**Additional File 1**

**Table S1.** Contents of schema therapy for high-functioning ASD

| **Phase 1**  **Psycho Educational & assessment**  **5 sessions** | ・Patients are given an explanation about the purpose of schema therapy and the flow structure of the 25 sessions  ・Patients receive feedback about “What is ASD?” and “What are the characteristics of my ASD?” based on various assessment tests and develop their own understanding of their condition  ・Patients learn what the secondary disabilities of ASD are  ・Patients understand the concepts used in schema therapy (ex. Early maladaptive schema and schema mode) |
| --- | --- |
| **Phase 2**  **Case conceptualization**  **Meet patient’s emotional needs**  **Schema mode change**  **10 sessions** | ・Patients understand how early maladaptive schemas and schema modes created by friction between their autistic traits, and their environment are creating difficulties in their daily lives using the schema therapy model  ・Through image techniques, patients will experience the fulfillment of core emotional needs (feeling relief, security etc.) that should have been naturally fulfilled during childhood  ・Patients will understand the characteristics of ASD, transform the schema modes that cause difficulties in their lives into an adaptive mode (healthy adult mode) that takes into account these characteristics |
| **Behavioral pattern braking Intervention phase**  **10 sessions** | ・By becoming conscious of behaviors that address the characteristics of ASD (healthy adult mode) and performing them, patients will practice reducing the difficulty they experience in their lives |
| **Follow-up**  **1 session**  **12 weeks after** | ・It will be determined if patients can make adaptations and live with ASD |
