# Supplementary material for: A preliminary study of schema therapy for young adults with high-functioning autism spectrum disorder: a single-arm, uncontrolled trial
Source: BMC Res Notes. 2021 Apr 29;14:158. doi: 10.1186/s13104-021-05556-1 (PMC8082897; doi:10.1186/s13104-021-05556-1)
Supplement: Supplementary file 2 — Additional file 2: Table S2. Sociodemographic data. [file 13104_2021_5556_MOESM2_ESM.docx]

**Additional File 2**

**Table S2**. Sociodemographic data

| Participant | Sex | Age | Education | Employment | Diagnosis  Time of diagnosis | Treatment before Intervention | Medication | Condition of pre-treatment | Condition of after-treatment |
| --- | --- | --- | --- | --- | --- | --- | --- | --- | --- |
| 1 | Female | 25 | Master | Student | ASD + OCD  At age 23 | Psychiatrist consultation | Not applicable | Nonattendance at school | Attendance at school |
| 2 | Male | 35 | High school | Unemployed | ASD + OCD  At age 35 | Not applicable | Not applicable | Unemployed | Go to work transition support |
| 3 | Male | 39 | Bachelor | Unemployed | ASD + OCD  At age 38 | Not applicable | Not applicable | Unemployed | Go to work transition support |
| 4 | Male | 22 | Bachelor | Student | ASD+DEP  At age 22 | Not applicable | Not applicable | Nonattendance at school | Attendance at school |
| 5 | Female | 21 | Bachelor | Student | ASD+DEP  At age 21 | Not applicable | Not applicable | Get in trouble with friends | Less trouble with friends |
| 6 | Male | 21 | Bachelor | Student | ASD + ADHD  At age 21 | Not applicable | Not applicable | Get in trouble with friends | Less trouble with friends |
| 7 | Female | 27 | Bachelor | Student | ASD + DEP  At age 25 | Psychiatrist consultation | Not applicable | Nonattendance at school | Go to work transition support |
| 8 | Male | 24 | Master | Student | ASD + DEP  At age 24 | Not applicable | Not applicable | Nonattendance at school | Go to work transition support |
| 9 | Female | 22 | Bachelor | Student | ASD+DEP  At age 22 | Not applicable | Not applicable | Nonattendance at school | Go to work transition support |
| 10 | Female | 32 | Bachelor | Part-timer | ASD + OCD  At age 22 for OCD, and age 32 for ASD | Psychiatrist consultation | Medicated for OCD | Get in trouble with family | Less trouble with family |

OCD, obsessive-compulsive disorder; DEP, depression; ADHD, attention deficit hyperactivity disorder; ASD, autism spectrum disorder
