# Supplementary material for: A preliminary study of schema therapy for young adults with high-functioning autism spectrum disorder: a single-arm, uncontrolled trial
Source: BMC Res Notes. 2021 Apr 29;14:158. doi: 10.1186/s13104-021-05556-1 (PMC8082897; doi:10.1186/s13104-021-05556-1)
Supplement: Supplementary file 3 — Additional file 3: Table S3. Changes in Scores of Young Schema Questionnaire (YSQ) before and after ST at follow-up. [file 13104_2021_5556_MOESM3_ESM.docx]

**Additional File 3**

**Table S3**. Changes in Scores of Young Schema Questionnaire (YSQ) before and after ST at follow-up.

|  | Pre | Post | Follow-up |  | Cohen's *d* | | |
| --- | --- | --- | --- | --- | --- | --- | --- |
| Variable | Mean (SD) | Mean (SD) | Mean (SD) | *F* | Pre vs post | Post vs follow-up | Pre vs follow-up |
| Total score | 314.40 (71.81)^a^ | 267.00 (82.15)^ab^ | 244.00 (68.30)^b^ | *F* (1.28, 11.51) = 15.01^**^ | 0.62 | 0.31 | 1.01 |
| Disconnection and rejection | 114.90(34.51)^ab^ | 96.00(30.81)^a^ | 86.20(26.53)^b^ | F(1.25,11.21) = 13.39^**^ | 0.58 | 0.34 | 0.93 |
| Impaired autonomy and performance | 121.50(26.95)^a^ | 102.40(35.05)^a^ | 92.20(29.35)^a^ | F(2,18) = 8.10^**^ | 0.61 | 0.32 | 1.04 |
| Impaired limits (Over-vigilance and inhibition) | 31.70(6.60)^a^ | 28.00(9.45)^ab^ | 27.70(6.02)^b^ | F(2,18) = 25.93^**^ | 0.45 | 0.04 | 0.63 |
| Overinhibition | 46.30(11.86)^a^ | 40.60(10.70)^a^ | 37.90(10.96)^a^ | F(2,18) = 6.45^**^ | 0.51 | 0.25 | 0.74 |
| Emotional Deprivation | 17.80 (7.84)^a^ | 15.00 (7.38)^a^ | 14.70 (5.38)^a^ | *F* (2, 18) = 2.46 | 0.37 | 0.05 | 0.46 |
| Abandonment / Instability | 16.80 (6.20)^a^ | 14.90 (7.85)^a^ | 13.30 (7.50)^a^ | *F* (2, 18) = 2.59 | 0.27 | 0.21 | 0.51 |
| Mistrust / Abuse | 16.00 (7.21)^a^ | 14.60 (6.95)^a^ | 13.50 (6.29)^a^ | *F* (1.22, 10.95) = 1.04 | 0.20 | 0.17 | 0.37 |
| Social Isolation / Alienation | 22.60 (5.32)^a^ | 18.60 (4.14)^a^ | 15.50 (3.95)^a^ | *F* (1.23, 11.11) = 8.09^*^ | 0.84 | 0.77 | 1.52 |
| Defectiveness / Shame | 20.20 (7.87)^a^ | 16.30 (6.65)^a^ | 14.50 (6.29)^a^ | *F* (1.16, 10.45) = 3.57 | 0.54 | 0.28 | 0.80 |
| Failure | 21.40 (6.20)^a^ | 15.80 (6.88)^a^ | 14.80 (6.07)^a^ | *F* (2, 18) = 9.00^**^ | 0.86 | 0.16 | 1.08 |
| Dependence / Incompetence | 20.60 (4.79)^a^ | 16.50 (6.98)^a^ | 14.90 (6.54)^a^ | *F* (1.1, 9.93) = 15.59^**^ | 0.69 | 0.24 | 1.00 |
| Vulnerability to Harm or Illness | 16.30 (5.12)^a^ | 13.80 (5.92)^a^ | 12.90 (6.06)^a^ | *F* (2, 18) = 2.89 | 0.45 | 0.15 | 0.61 |
| Enmeshment / Undeveloped Self | 14.40 (4.40)^a^ | 12.30 (4.83)^a^ | 10.80 (4.32)^a^ | *F* (2, 18) = 3.01 | 0.46 | 0.33 | 0.83 |
| Subjugation | 14.80(5.12)^a^ | 14.10 (5.09)^a^ | 12.40 (3.53)^a^ | *F* (2, 18) = 1.30 | 0.14 | 0.39 | 0.55 |
| Self-Sacrifice | 10.80(4.71)^a^ | 9.30(3.53)^a^ | 9.60(3.75)^a^ | *F* (2, 18) = 1.17 | 0.36 | 0.08 | 0.28 |
| Emotional Inhibition | 19.20(5.88)^a^ | 16.10(3.35)^a^ | 13.70(3.80)^a^ | *F* (1.28, 11.48) = 6.44^*^ | 0.65 | 0.67 | 1.11 |
| Unrelenting Standard / Hypercriticalness | 17.00(4.19)^a^ | 16.80(4.96)^a^ | 15.10(4.33)^a^ | *F* (2, 18) = 2.11 | 0.04 | 0.37 | 0.45 |
| Entitlement / Grandiosity | 14.50(3.78)^a^ | 13.30(4.42)^a^ | 13.60(3.66)^a^ | *F* (2, 18) = 0.82 | 0.29 | 0.07 | 0.24 |
| Insufficient Self-Control / Self-Discipline | 17.20(7.24)^a^ | 15.00(5.33)^a^ | 13.10(3.75)^a^ | *F* (2, 18) = 3.25 | 0.35 | 0.41 | 0.71 |
| Approval-Seeking / Recognition-Seeking | 17.20(3.97)^a^ | 14.70(6.48)^a^ | 14.10(4.63)^a^ | *F* (2, 18) = 3.91^*^ | 0.47 | 0.11 | 0.72 |
| Negativity / Pessimism | 19.10(6.81)^a^ | 15.40(5.95)^a^ | 14.30(5.48)^a^ | *F* (1.14, 10.24) = 5.28^*^ | 0.58 | 0.19 | 0.78 |
| Punitiveness | 18.50(5.84)^a^ | 14.50(4.12)^a^ | 13.20(4.24)^a^ | *F* (2, 18) = 11.77^**^ | 0.79 | 0.31 | 1.04 |
| ^**^p<0.01. ^*^p <0.05 |  |  |  |  |  |  |  |

Note: The same letters were not significantly different in pairwise comparisons.
