# Supplementary material for: A preliminary study of schema therapy for young adults with high-functioning autism spectrum disorder: a single-arm, uncontrolled trial
Source: BMC Res Notes. 2021 Apr 29;14:158. doi: 10.1186/s13104-021-05556-1 (PMC8082897; doi:10.1186/s13104-021-05556-1)
Supplement: Supplementary file 4 — Additional file 4: Table S4. Changes in Schema Mode Inventory (SMI) before and after ST at follow-up. [file 13104_2021_5556_MOESM4_ESM.docx]

**Additional File 4**

**Table S4.** Changes in Schema Mode Inventory (SMI) before and after ST at follow-up.

| SMI |  |  |  |  |  |  |  |
| --- | --- | --- | --- | --- | --- | --- | --- |
|  | pre | post | follow-up |  | Cohen's *d* | | |
| Variable | Mean (SD) | Mean (SD) | Mean (SD) | *F* | Pre-post | Post-follow up | Pre-follow up |
| Adaptive modes　total | 50.90 (11.99)^a^ | 59.50 (13.11)^a^ | 62.40 (14.03)^a^ | *F*(1.28,11.51) = 10.12^**^ | 0.69 | 0.21 | 0.88 |
| Maladaptive modes total | 313.80(74.53)^a^ | 267.40 (71.28)^ab^ | 215.20 (48.10)^bc^ | *F*(2,18) = 15.78^**^ | 0.64 | 0.86 | 1.57 |
| Vulnerable child | 38.80(11.83)^a^ | 30.50 (10.56)^a^ | 25.60 (10.66)^a^ | *F*(2,18) = 8.15^**^ | 0.74 | 0.46 | 1.17 |
| Angry child | 28.90 (9.40)^a^ | 26.40 (8.41)^a^ | 20.10 (5.78)^a^ | *F*(2,18) = 4.47^*^ | 0.28 | 0.87 | 1.13 |
| Enraged child | 22.30 (5.29)^a^ | 18.40 (8.00)^ab^ | 13.70 (3.86)^bc^ | *F*(2,18) = 12.59^**^ | 0.58 | 0.75 | 1.86 |
| Impulsive child | 28.00 (10.84)^a^ | 22.80 (9.82)^a^ | 17.30 (4.45)^a^ | *F*(2,18) = 9.64^**^ | 0.50 | 0.72 | 1.29 |
| Undisciplined child | 20.00 (7.56)^a^ | 16.30 (6.17)^ab^ | 13.80 (5.03)^bc^ | *F*(2,18) = 9.52^**^ | 0.54 | 0.45 | 0.97 |
| Happy child | 20.50 (7.09)^a^ | 25.40 (8.32)^a^ | 25.60 (8.42)^a^ | *F*(2,18) = 5.30^*^ | 0.63 | 0.02 | 0.66 |
| Compliant surrenderer | 21.30 (7.35)^a^ | 18.90 (7.36)^a^ | 16.40 (6.96)^a^ | *F*(1.24,11.15) = 9.11^**^ | 0.33 | 0.35 | 0.69 |
| Detached protector | 32.10 (12.49)^a^ | 25.30 (8.14)^a^ | 21.10 (7.36)^a^ | *F*(2,18) = 8.07^**^ | 0.65 | 0.54 | 1.07 |
| Detached self-soothing | 13.00 (4.55)^a^ | 10.60 (4.95)^a^ | 10.70 (5.54)^a^ | *F*(1.3,11.67) = 3.16 | 0.51 | 0.02 | 0.45 |
| Self-aggrandizer | 26.30 (5.85)^a^ | 23.50 (6.29)^a^ | 17.20 (4.44)^a^ | *F*(2,18) = 9.35^**^ | 0.46 | 1.16 | 1.75 |
| Bully attack | 20.40 (7.20)^a^ | 20.40 (5.74)^a^ | 15.50 (3.98)^a^ | *F*(2,18) = 3.26 | 0.00 | 0.99 | 0.84 |
| Punitive parent | 31.10 (13.63)^a^ | 26.30 (13.10)^a^ | 21.00 (8.38)^a^ | *F*(2,18) = 7.18^**^ | 0.36 | 0.48 | 0.89 |
| Demanding parent | 31.60 (8.15)^a^ | 28.00 (10.03)^ab^ | 22.80 (7.79)^bc^ | *F*(2,18) = 16.27^**^ | 0.39 | 0.58 | 1.10 |
| Healthy adult | 30.40 (7.21)^a^ | 34.10 (6.66)^a^ | 36.80 (7.52)^a^ | *F*(1.26,11.34) = 18.80^**^ | 0.53 | 0.38 | 0.87 |

^**^p<0.01. ^*^p<0.05

SMI: Schema Mode Inventory

Note: The same letters are not significantly different in pairwise comparisons.
